# *Terc* Gene Cluster Variants Predict Liver Telomere Length in Mice

**DOI:** 10.3390/cells10102623

**Published:** 2021-10-01

**Authors:** Dana Zeid, Sean Mooney-Leber, Laurel R. Seemiller, Lisa R. Goldberg, Thomas J. Gould

**Affiliations:** 1 Department of Biobehavioral Health, Penn State University, University Park, PA 16802, USA; lrs5307@psu.edu (L.R.S.); lrg33@psu.edu (L.R.G.); tug70@psu.edu (T.J.G.); 2 Department of Psychology, University of Wisconsin-Stevens Point, Stevens Point, WI 54481, USA; smooneyl@uwsp.edu

**Keywords:** telomeres, genetic, nicotine, aging, Terc, Mynn, Lrriq4, Lrrc31, cancer

## Abstract

Variants in a gene cluster upstream-adjacent to *TERC* on human chromosome 3, which includes genes *APRM*, *LRRC31*, *LRRC34* and *MYNN*, have been associated with telomere length in several human populations. Currently, the mechanism by which variants in the *TERC* gene cluster influence telomere length in humans is unknown. Given the proximity between the *TERC* gene cluster and *TERC* (~0.05 Mb) in humans, it is speculated that cluster variants are in linkage disequilibrium with a *TERC* causal variant. In mice, the *Terc* gene/*Terc* gene cluster are also located on chromosome 3; however, the *Terc* gene cluster is located distantly downstream of *Terc* (~60 Mb). Here, we initially aim to investigate the interactions between genotype and nicotine exposure on absolute liver telomere length (aTL) in a panel of eight inbred mouse strains. Although we found no significant impact of nicotine on liver aTL, this first experiment identified candidate single nucleotide polymorphisms (SNPs) in the murine *Terc* gene cluster (within genes *Lrrc31*, *Lrriq4* and *Mynn*) co-varying with aTL in our panel. In a second experiment, we tested the association of these *Terc* gene cluster variants with liver aTL in an independent panel of eight inbred mice selected based on candidate SNP genotype. This supported our initial finding that *Terc* gene cluster polymorphisms impact aTL in mice, consistent with data in human populations. This provides support for mice as a model for telomere dynamics, especially for studying mechanisms underlying the association between *Terc* cluster variants and telomere length. Finally, these data suggest that mechanisms independent of linkage disequilibrium between the *Terc*/*TERC* gene cluster and the *Terc*/*TERC* gene mediate the cluster’s regulation of telomere length.

## 1. Introduction

Telomeres are repetitive nucleotide sequences located at both ends of eukaryotic linear chromosomes (see [1,2] for review). Telomeres represent an adaptive solution to the “end-replication problem” associated with replication of linear chromosomes. Replication of double-stranded, linear chromosomes requires simultaneous extension of both strands in the 5′ to 3′ direction via DNA polymerase, with new nucleotides added onto an available 3′-OH group. Thus, synthesis of the new strand by DNA polymerase is continuous only on one strand (leading strand). Replication on the other strand (lagging) is discontinuous and synthesized in a series of adjacent fragments, wherein primases insert primers and DNA polymerase elongates the sequence in the 5′ to 3′ direction. Primers are then removed and fragments are ligated. However, when the last primer is removed from the lagging strand end, there is no adjacent fragment with an available 3′-OH group for extension by DNA polymerase. Thus, each subsequent cell division results in a progressively shorter sequence on the lagging strand. Repetitive telomere sequences located at the ends of chromosomes buffer coding DNA from shortening during replication. Because telomeres shorten with each cell cycle, they have been quantified as a proxy for aging [3].

The enzyme telomerase can extend the telomere sequence, thus delaying cellular senescence. However, in humans, telomerase is only active in select, highly proliferating cell populations, such as gametes and cancer cells [4]. In mice, low telomerase activity is also detectable in most adult organs [5]. This species difference is theorized to reflect differential adaptive strategies surrounding telomere dynamics. Specifically, it is thought that telomerase inactivation in humans represents an evolutionary trade-off to reduce cancer incidence in our relatively large, long-living species (see [6] for review).

Telomerase is a ribonucleoprotein whose domains include a reverse transcriptase, encoded by the gene *TERT* (mouse ortholog *Tert*) and an RNA template, encoded by the gene *TERC* (mouse ortholog *Terc*). The telomere sequence is identical between mice and humans and the products of these telomerase genes are thought to function similarly between the species, albeit with different physiological distribution and activity rates (see [6] for review).

Telomere length is highly heritable in human populations as a result of sequence variation in genomic networks regulating telomere length [7,8]. Variants in a gene cluster upstream-adjacent to *TERC* on human chromosome 3, which includes genes *APRM*, *LRRC31*, *LRRC34* and *MYNN*, have been found to associate with telomere length [9,10,11,12,13,14], as well as related diseases, such as cancer and diabetes [9,11,15,16,17], in several human populations by multiple independent analyses.

**Figure 1:**
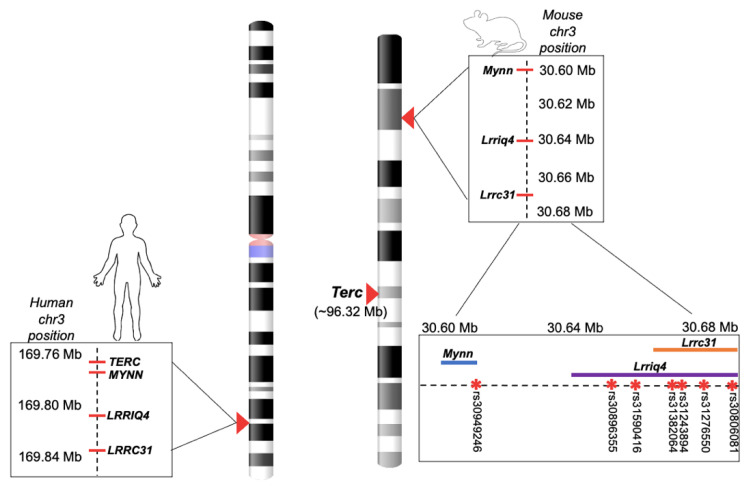
*TERC*/*Terc* gene and *TERC*/*Terc* gene cluster (*Lrrc31*, *Lrriq4*, *Mynn*) in human (**left**) and mouse (**right**) chromosome 3. Candidate SNPs for telomere length within mouse *Terc* gene cluster marked on expanded chromosome 3 view (**bottom right**).

Genes *APRM*, *LRRC31*, *LRRC34*, *MYNN* and their mouse orthologs are found in a conserved synteny block located on chromosome 3 of both species (queried using the JAX Synteny and Synteny Portal online tools; [18,19]). In humans, this block is directly adjacent to the *TERC* gene, while, in mice, the *Terc* gene is located more distantly from this gene cluster (see Figure 1). Currently, the mechanism by which variants in the *TERC* gene cluster influence telomere length in humans is unknown. Notably, few studies have directly associated telomere length with polymorphisms within the *TERC* gene itself. Only the *TERC* polymorphism rs2293607 has been associated with telomere length in human genomic analyses and this SNP is strongly correlated with *MYNN* variants [11].

Due to the close proximity between the *TERC* gene cluster and *TERC* (~0.05 Mb) in humans, it has been speculated that genes in this cluster may regulate *TERC* expression [13], or that they are in linkage disequilibrium with a true causal variant within or nearer to *TERC* [11]. In the mouse genome, the *Terc* gene and *Terc* gene cluster are also both located on chromosome 3; however, the *Terc* gene cluster is located distantly downstream of *Terc* (~60 Mb) in mice. If the *Terc* gene cluster also regulates telomere length in mice, this species may represent a valuable tool for investigating mechanisms underlying the regulation of telomere length by *Terc* gene cluster variants in a model that minimizes the confounding effects of linkage disequilibrium.

To our knowledge, no mouse studies have associated variants in the *Terc* gene cluster with telomere length. Inbred mouse strain panels are a useful tool for modeling genomic variance at the level of discovery research in animal subjects. However, uncertainty has been expressed regarding the suitability of mice, especially inbred mice, for telomere research. It has been found that laboratory mouse (Mus musculus) telomeres are generally longer than human telomeres [20] and inbreeding to generate inbred mouse strains may result in spontaneous telomere elongation [21]. On the other hand, it has also been shown that shortened telomeres associate with aging, cancer and related pathology in both mice and humans [22,23,24]. Further, despite the fact that telomerase is more broadly active in somatic cells of adult mice versus humans, telomerase upregulation is seen in both human and mouse tumors [25,26].

The current study initially aimed to investigate the effect of the interactions between genotype and nicotine exposure at one chronic dose on absolute liver telomere length in a panel of inbred mouse strains. Although no significant impact of nicotine exposure on absolute liver telomere length was found, this first experiment identified candidate variants in the murine *Terc* gene cluster (SNPs located in *Lrrc31*, *Lrriq4* and *Mynn*) that co-varied with telomere length in our panel. In a second experiment, we tested the effect of these *Terc* gene cluster variants on absolute liver telomere length in an independent panel of inbred mouse strains selected based on genotype at candidate SNPs within the chromosome 3 cluster. This second experiment supported our finding that polymorphisms in the *Terc* gene cluster impact telomere length in inbred mouse strains, replicating findings in human populations. These findings provide support for inbred mouse strains as a model for telomere dynamics, especially for studying mechanisms underlying the association between *Terc* gene cluster variants and telomere length.

## 2. Materials and Methods

### 2.1. Experiment 1

#### 2.1.1. Experiment 1: Overview

The initial aim of Experiment 1 was to test effects of nicotine exposure on liver telomere length in a panel of inbred mouse strains. Animals were a part of a larger project testing effects of nicotine exposure and genetic background on fear conditioning. Thus, animals were previously exposed to a cued/contextual fear conditioning paradigm (ending one day prior to euthanasia). Subjects were also exposed to 18 mg/kg/day nicotine or saline over a period of 12 days via subcutaneous osmotic minipump. Liver tissue for telomere length quantification was dissected three days following removal of drug or vehicle. Fear conditioning and drug exposure methodology can be found in Supplementary Materials.

#### 2.1.2. Experiment 1: Subjects

The subjects were adult (10–13 weeks at time of liver dissection), male mice of eight inbred mouse strains: 129S2/SvPasCrl, 129S4/SvJaeJ, 129S8/SvEvNimrJ, BTBR T+ Itpr3tf/J, C57BL/6J, MA/MyJ, NZB/BINJ and SM/J (n = 9 per treatment group per strain, all strains aside from 129S2/SvPasCrl purchased from Jackson Laboratory, Bar Harbor, ME, USA; 129S2/SvPasCrl purchased from Charles River, Wilmington, MA, USA). All mice were group-housed in the same colony room with a 12 h light/dark cycle and ad libitum access to food and water. All procedures were performed in accordance with the NIH Guide for the Care and Use of Laboratory Animals and were approved by the Pennsylvania State University IACUC committee.

#### 2.1.3. Experiment 1: Liver Dissection and DNA Extraction

Liver tissue from the left lobe was dissected immediately following euthanasia by cervical dislocation, which occurred three days after osmotic minipump removal. Dissections were performed at room temperature and dissected tissue was stored at −80 °C. DNA was extracted from liver tissue using the Qiagen DNeasy Blood and Tissue Kit (Hilden, Germany) according to the manufacturer’s instructions. DNA purity was assessed using 260/280 and 260/230 absorbance ratio readings on NanoDrop 2000 (Thermo Scientific; Wilmington, DE, USA). Liver DNA concentration was quantified using the Quant-iT PicoGreen dsDNA assay kit (ThermoFisher; Waltham, MA, USA). For Experiment 1, Picogreen DNA quantification was performed by the Penn State Biomarker Core Laboratory. Samples were read on the Synergy 2 Multi-Mode Plate Reader (Biotek; Winooski, VT, USA) at an excitation wavelength of 485 nm and an emission wavelength of 528 nm. All samples were diluted to a concentration of 1 ng/µL for subsequent telomere length measurement.

#### 2.1.4. Experiment 1: Telomere Length Quantification

Absolute telomere length (aTL) was measured using a quantitative PCR method adapted from O’Callaghan and Fenech [27] (originally adapted from T/S ratio method by Cawthon [28]). Briefly, this assay utilizes an oligomer telomere standard ladder alongside quantification of genome copy number to calculate aTL. The single-copy mouse gene *B2m* was selected for genome copy number quantification [29,30]. Separate PCR reactions of DNA from the same sample were run to quantify total telomere content and genome-copy number, respectively. Telomere length quantification was performed by the Penn State Biomarker Core Laboratory for Experiment 1. The PCR reaction was run as follows: a 2 min hold at 50 °C (UNG activation) followed by a 15 min hold at 95 °C (genome denaturation and hot start Taq activation), followed by 45 cycles of 15 s at 95 °C, then 1 min at 60 °C. The final PCR master mix consisted of 6 µL of 1 ng/µL sample DNA, 10 µL of 2× Quantitect SYBR Green (Qiagen, Hilden, Germany), 0.2 µL of 10 µM forward primer, 0.2 µL of 10 µM reverse primer, 0.2 µL of 1 U/µL uracil-DNA glycosylase (UNG, ThermoFisher, Waltham, MA, USA) and 3.4 µL of molecular-grade water. Real-time PCR was run in triplicate on the Rotor-Gene Q (Qiagen, Hilden, Germany). Samples were balanced across PCR plates by strain and treatment.

Primers and standard oligomers were ordered as custom sequences from IDT (Integrated DNA Technologies; Coralville, IA, USA). The telomere primer sequences were as follows: forward primer 5′-CGGTTTGTTTGGGTTTGGGTTTGGGTTTGGGTTTGGGTT-3′, reverse primer 5′-GGCTTGCCTTACCCTTACCCTTACCCTTACCCTTACCCT-3′. The B2m primer sequences were as follows: forward primer 5′-CTCGGTGACCCTGGTCTTTC-3′, reverse primer 5′-CACTCACTCTGGATAGCATAC-3′. For Experiment 1, PCR plate normalization was initially performed using a standard DNA sample included on all plates. aTL per genome was calculated as total telomere content per sample/genome copy number per sample. aTL *per telomere* was calculated as aTL per genome/80 (total number of telomeres in diploid mouse genome). Note that the reported data are in *per telomere* units, calculated as mean aTL per diploid genome divided by 80 (the total number of telomeres in the diploid mouse genome).

#### 2.1.5. Experiment 1: SNP Query and Genotyping

**Figure 2:**
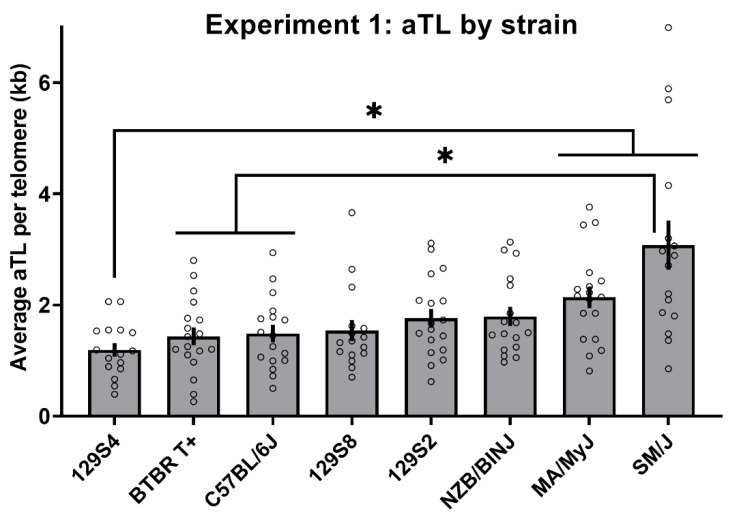
Average liver aTL per telomere (kb) in Experiment 1 inbred mouse strains. * Indicates significant strain differences at a Games–Howell corrected significance threshold of 0.05. Unfilled circles indicate individual datapoints per strain. n = 16–18 per strain.

An SNP query was performed in Experiment 1 strains to identify genotypes that segregated with telomere length. The Mouse Phenome Database (MPD) SNP data retrieval utility tool [31] (phenome.jax.org) was used to query available strain genotype data in 20 candidate genes previously shown to associate with telomere length (through known biological pathways or human GWAS studies queried using the EMBL-EBI GWAS catalog, www.ebi.ac.uk/gwas (accessed on 11 December 2020)). These genes were *Actrt3* (human homolog *APRM1*), *Acyp2*, *Bicd1*, *Cast*, *Csnk2a2*, *Dcaf4*, *Dkc1*, *Lrrc31*, *Lrrc34*, *Lrriq4*, *Mynn*, *Ncr1*, *Parp1*, *Rtel1*, *Stn1*, *Terc*, *Terf1*, *Tert*, *Tgm1* and *Tnr*. The query was performed using SM/J (the longest liver aTL strain from Experiment 1) as the reference strain. The query identified seven candidate SNPs in the *Terc* gene cluster that covaried with telomere length in our strain panel. Specifically, the two strains with the longest liver telomeres (SM/J and MA/MyJ, see Figure 2) among this initial panel had unique alleles at rs31382064, rs31243894, rs31276550 and rs30806081 (annotated to *Lrrc31*), rs30896355 and rs31590416 (annotated to *Lrriq4*) and rs30949246 (annotated to *Mynn*) (see Figure 1, bottom right, for candidate SNP localization on mouse chromosome 3). Because genotype information was unavailable for two of the tested strains (129S2/SvPasCrl and 129S8/SvEvNimrJ), these strains were genotyped using Sanger sequencing at 6 of 7 of the candidate SNPs (see Supplementary Materials). This genotyping confirmed unique alleles at all seven candidate SNPs in SM/J and MA/MyJ compared to the other tested strains. Genealogical relationships between the tested strains were also referenced using the comprehensive inbred mouse genealogy mapping published by Beck et al. [32], which indicated that SM/J and MA/MyJ were not more closely related than other strains within the panel.

#### 2.1.6. Experiment 1: Statistical Analyses

Statistical analyses for Experiments 1 and 2 were performed using the SPSS software, v26 (IBM, Armonk, NY, USA). Outliers, defined as datapoints ±2 SDs from the strain mean, were first filtered from the Experiment 1 dataset (8 total datapoints removed). The effects of strain and nicotine treatment were initially tested in a mixed-effects ANOVA with strain and treatment as between-subjects factors and plate as a random factor. This analysis was followed by a one-way ANOVA with strain as a between-subjects factor and plate as a random factor. Plate was included as a factor to statistically control for random plate-to-plate variation. The White test for heteroscedasticity [33] was used to test for the assumption of dependent variable homoscedasticity. For analyses in which the ANOVA assumption of homoscedasticity was violated, main and interaction effects were verified using a non-parametric procedure (proportional odds ordinal logistic regression, a ranked data model [34]). Strain means were compared using Games–Howell corrected post hoc tests.

### 2.2. Experiment 2

#### 2.2.1. Experiment 2: Overview

Experiment 1 identified SNPs in *Mynn*, *Lrriq4* and *Lrrc31* as candidate regulators of liver telomere length in inbred mice. Experiment 2 was designed to test the allelic effect of these SNPs in an independent panel of inbred mouse strains selected based on genotype at candidate SNPs. This experiment also included female subjects in order to test for potential sex effects on telomere length in inbred mouse strains.

#### 2.2.2. Experiment 2: Strain Selection

Genotype information at candidate SNPs was queried using the MPD SNP data retrieval utility tool (phenome.jax.org [31], accessed 11 December 2020). Specifically, a dataset including genotype data for a large collection of inbred mice (“Broad2” dataset) was used for the selection of four strains with the “long” (SM/J and MA/MyJ) allele at all seven candidate SNPs and four strains with the “short” allele at all seven candidate SNPs. Any missing genotype information in candidate SNPs was confirmed using the “Sanger4” SNP dataset, also available through the MPD SNP query tool. Within the dataset, we identified 43 strains with the “short” allele at all candidate SNPs, 26 strains with mixed short and long alleles and 13 strains with the “long” allele at all candidate SNPs. A total of 4 of the 43 “short” allele strains (129X1/SvJ, BALB/cJ, C57BL10/J and FVB/nJ) and 4 of the 13 “long” allele strains (A/J, C3H/HeJ, CBA/J, NOD/ShiLtJ) were selected, prioritizing distant genealogical relationships in strains currently available for purchase (based on the comprehensive inbred mouse genealogy mapping published by Beck et al. [32]).

#### 2.2.3. Experiment 2: Subjects

The subjects were adult (aged 7–9 weeks at time of liver dissection) male and female mice of eight inbred mouse strains: 129X1/SvJ, BALB/cJ, C57BL10/J, FVB/nJ (“short” allele strains) and A/J, C3H/HeJ, CBA/J, NOD/ShiLtJ (“long” allele strains) (n = 7 per sex per strain, with the exception of C57BL/10J, which had only 4 females; Jackson Laboratory, Bar Harbor, ME, USA). Mice were group-housed in the same colony room with a 12 h light/dark cycle and ad libitum access to food and water. Subjects were acclimated to the colony room over a seven-day period following their arrival, after which liver dissections were performed. For Experiment 2, subjects did not receive any experimental manipulation prior to euthanasia. All procedures were performed in accordance with the NIH Guide for the Care and Use of Laboratory Animals and were approved by the Pennsylvania State University IACUC committee.

#### 2.2.4. Experiment 2: Liver Dissection and DNA Extraction

Liver tissue from the left lobe was dissected immediately following CO_2_ euthanasia. Dissections were performed at room temperature and dissected tissue was stored at −80 °C. DNA extractions and DNA quality/quantity assessment were performed using the same methodology detailed in Experiment 1. All DNA samples were diluted to a concentration of 1.5 ng/µL for subsequent telomere length measurement.

#### 2.2.5. Experiment 2: Telomere Length Quantification

For Experiment 2, absolute telomere length (aTL) was measured using methods nearly identical to those used in Experiment 1. Because telomere length quantification was performed by independent experimenters for Experiment 1 and Experiment 2, there were some minor differences in methodology: First, real-time PCR was run in triplicate on the Applied Biosystems 7500 Fast Real-Time PCR thermal cycler (Waltham, MA, USA) for Experiment 2. Second, Experiment 2 DNA samples used for real-time PCR were slightly more concentrated (1.5 ng/µL). Finally, raw data (not normalized to a plate control) were used for Experiment 2 statistical analyses. Note that another form of plate normalization, the inclusion of plate as a random statistical factor, was still performed for Experiment 2.

#### 2.2.6. Experiment 2: Statistical Analyses

Experiment 2 outliers, defined as datapoints ±2 SDs from the strain mean, were first filtered from the dataset (6 total datapoints removed). The effects of the SNP group (“long” versus “short” genotype at chromosome 3 gene cluster candidate variants) and sex were initially tested in a mixed-effects ANOVA with SNP group and sex as between-subjects factors and plate as a random factor. Plate was included as a factor to statistically control for random plate-to-plate variation. This analysis was followed by a one-way ANOVA with SNP group as a between-subjects factors and plate as a random factor. The White test for heteroscedasticity [33] was used to test for the assumption of dependent variable homoscedasticity.

## 3. Results

For Experiment 1, a mixed-effects ANOVA of average aTL per telomere with strain and treatment as between-subjects factors and plate as a random factor revealed a significant main effect of strain [F(7,112) = 13.96, *p* < 0.001] and a significant random effect of plate [F(8,112) = 18.74, *p* < 0.001], but no significant effect of nicotine treatment (*p* = 0.38) and no significant interaction between strain and treatment (*p* = 0.89; see Figure S1 for data by treatment group). A follow-up, mixed-effects ANOVA with strain as a between-subjects factor and plate as a random factor was then run, which revealed a significant main effect of strain [F(7,120) = 14.42, *p* < 0.001] and a significant random effect of plate [F(8,120) = 19.86, *p* < 0.001]. However, the White test for heteroscedasticity indicated that the assumption of homogeneity was violated (*p* < 0.001). Thus, the main effect of strain was confirmed using a non-parametric procedure (proportional odds ordinal logistic regression; Wald chi-square = 31.96, *p* < 0.001; Figure 2). Games–Howell post hoc indicated that SM/J and MA/MyJ aTL strain means were significantly greater than those of 129S4/SvJaeJ (GH corrected *p* < 0.05). The SM/J aTL strain mean was also significantly greater than that of BTBR T+ Itpr3tf/J and C57BL/6J (GH corrected *p* < 0.05).

An SNP query of candidate genes previously shown to associate with telomere length was performed using Experiment 1 strains to identify genotypes that segregated with telomere length (see Methods Section 2.1.5 for SNP query details). The query identified seven candidate SNPs in the *Terc* gene cluster that covaried with telomere length in our strain panel. Specifically, the two strains with the longest liver aTL (SM/J and MA/MyJ; see Figure 2) among the Experiment 1 panel had unique alleles at rs31382064, rs31243894, rs31276550 and rs30806081 (located within *Lrrc31*), rs30896355 and rs31590416 (located within *Lrriq4*) and rs30949246 (located within *Mynn*). Experiment 2 strains were chosen based on allele at these candidate SNPs.

**Figure 3:**
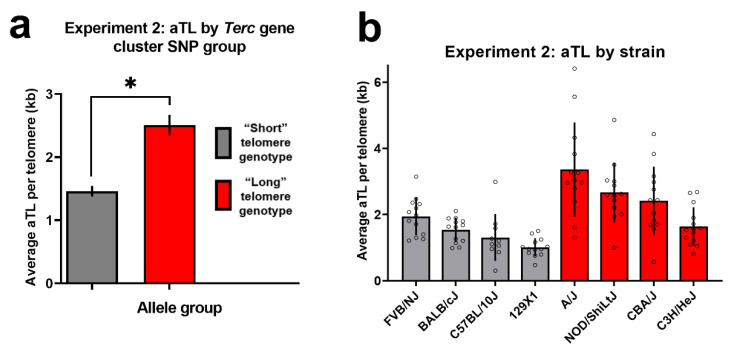
(**a**) Average liver aTL per telomere (kb) in Experiment 2 inbred mouse strains, by genotype group. “Short” genotype n = 49; “Long” genotype n = 53. * Indicates significant main effect at ANOVA significance threshold of 0.05. (**b**) Average liver aTL per telomere (kb) in Experiment 2 inbred mouse strains, shown by strain. Unfilled circles indicate individual datapoints per strain. n = 10–14 per strain.

For Experiment 2, a mixed-effects ANOVA of average aTL per telomere with SNP group (“short” vs. “long” allele at all seven candidate SNPs identified in Experiment 1), sex as between-subjects factors and plate as a random factor revealed a significant main effect of SNP group [F(1,92) = 36.70, *p* < 0.001] and a significant random effect of plate [F (6,92) = 5.20, *p* < 0.001], but no significant effect of sex (*p* = 0.48; see Figure S2 for data by sex) and no significant interaction between SNP group and sex (*p* = 0.80). A follow-up, mixed-effects ANOVA collapsed across sex, with strain as a between-subjects factor and plate as a random factor was then run, which revealed a significant main effect of SNP group [F(1,94) = 36.80, *p* < 0.001; Figure 3] and a significant random effect of plate [F(6,94) = 5.40, *p* < 0.001]. The White test for heteroscedasticity indicated that the assumption of homogeneity was satisfied (*p* > 0.05).

## 4. Discussion

Here, we found that variants in the *Terc* gene cluster (within genes *Lrrc31*, *Lrriq4* and *Mynn*) predicted telomere length in two independent inbred mouse strain panels. These data replicate findings in several human cohorts, which have identified the *Terc* gene cluster as a key candidate regulator of telomere length. These data reinforce the importance of this gene cluster in the regulation of telomere length across multiple mammalian species. This is the first study to our knowledge demonstrating the association between this genomic region and telomere length in mice. In humans, SNPs within this cluster are the genomic predictors most consistently associated with telomere length and related disease, despite limited knowledge on the mechanism underlying this association [9,10,11,12,13,14,15,16,17]. Elaboration of this association in an animal model may thus permit critical mechanistic research, in addition to promoting understanding of between-species telomere dynamics.

Both the *Terc*/*TERC* gene and *Terc*/*TERC* gene cluster are located on chromosome 3 in humans and mice. *TERC* and the *TERC* gene cluster are directly adjacent in humans (~0.05 Mb apart). On the other hand, in mice, the *Terc* gene is well removed from the *Terc* gene cluster (~60 Mb apart). Here, we found that genomic variants within the *Terc* gene cluster (in genes *Lrrc31*, *Lrriq4* and *Mynn*) also predict liver telomere length in mice. Thus, inbred mice may represent a valuable tool for investigating mechanisms underlying regulation of telomere length by *Terc* gene cluster variants in a model that minimizes the confounding effects of linkage disequilibrium.

Importantly, we also noted that segregation of liver telomere length by the SNP group (“long” versus “short” *Terc* cluster alleles) in Experiment 2 was imperfect. That is, there was some overlap in liver telomere length between the genotype groups (see Figure 3b). This variation is to be expected given that polymorphisms interact within the broader genomic context of a given mouse strain. Future work may aim to further characterize factors leading to inbred mouse strain differences in telomere length. The individual datapoints displayed in Figure 2 and Figure 3b demonstrate the degree of variability in telomere length between animals. This is consistent with previous findings that mouse telomere are hypervariable in length, even within an individual [35]. An additional important area of inquiry within this field is the biological impacts of telomere length in inbred mice. Here, we noted no consistent phenotypes related to cancer or aging that segregated with *Terc* cluster genotype or telomere length using public information on the tested strains (known strain phenotype information available through suppliers and a preliminary query in the MPD outlier phenotype tool, data not shown). However, as discussed previously, telomeres may take on different adaptive functions in humans versus mice [6]. Thus, consistent phenotypes correlated with telomere length in mice may be unknown at this time.

One notable limitation of this work is that these findings do not definitively eliminate the possibility that the identified gene cluster links to *Terc* function through linkage disequilibrium or through regulation of *Terc* function. The distance between *Terc* and the cluster on mouse chromosome 3 minimizes the likelihood of linkage disequilibrium between the gene and cluster, but cluster genes may still regulate *Terc* functioning through an unidentified pathway. Preliminary assessment of potential gene interactions through the Ingenuity Pathway Analysis “Interaction Network” search tool revealed no direct interactions between *Terc* and *Lrrc31*, *Lrriq4*, or *Mynn* (content version 65367011 [36]). Future research may aim to clarify these questions through full gene sequencing and comprehensive profiling of potential biological interactions between *Terc* and other telomere-associated genes with *Lrrc31*, *Lrriq4* and *Mynn*.

The current study also tested effects of chronic nicotine exposure at one dose on liver telomere length in inbred mouse strains. We found no significant impacts of chronic nicotine exposure on liver telomere length, which is inconsistent with at least one study finding that germ cell telomeres were shortened in mice exposed to nicotine [37]. However, telomere dynamics differ between liver and germ cells, and nicotine exposure at only one dose was tested here. It is possible that different nicotine doses or exposure conditions impact liver telomere length in mice, or that nicotine’s impacts on telomere length are cell-type specific. Another limitation of this drug exposure design was that telomeres were only quantified at one timepoint. Telomere dynamics over time are also consequential to biological functioning, as these likely fluctuate as a function of other environmental factors, as well as age [38]. Thus, an age-dependent effect of nicotine on telomere length is also possible. It should also be noted that nicotine exposure models in mice are not directly comparable to analyses in human smokers, whose exposure is longer and includes the non-nicotine chemical constituents of tobacco smoke.

Here, we found no effect of sex on telomere length in inbred mouse strains tested in Experiment 2. Sex differences in telomere length have been identified in humans and other mammals [39,40], although these studies have typically sampled leukocytes. Thus, it is possible that sex differences in telomere length are restricted to certain cell populations. Other explanations may include relatively low power to detect these differences in the current sample, or simply that there are no sex differences in liver telomere length within the tested inbred mouse strains.

In summary, this study demonstrates the association between variants in the *Terc* gene cluster and telomere length in a non-human model. These findings support identification of the *Terc*/*TERC* gene cluster (specifically genes *Lrrc31*, *Lrriq4*, *Mynn* and their human homologs) as key regulators of telomere length. These data further suggest that mechanisms independent of linkage disequilibrium between the *Terc*/*TERC* gene cluster and the *Terc*/*TERC* gene are responsible for the *Terc*/*TERC* gene cluster’s association with telomere length.

## Data Availability

The data presented in this study are openly available on the Zenodo repository at https://doi.org/10.5281/zenodo.5541771 (accessed on 11 December 2020).

## Supplementary Materials

The following are available online at https://www.mdpi.com/article/10.3390/cells10102623/s1, Figure S1: Experiment 1 aTL by strain and drug treatment, Figure S2: Experiment 2 aTL by strain and sex, Supplementary Methods (Fear conditioning, Drug exposure and Genotyping [41,42]), Table S1: Genotyping primer information.
