# Supplementary material for: Terc Gene Cluster Variants Predict Liver Telomere Length in Mice
Source: Cells. 2021 Oct 1;10(10):2623. doi: 10.3390/cells10102623 (PMC8533930; doi:10.3390/cells10102623)
Supplement: Supplementary file 1 [file cells-10-02623-s001.zip › cells-1377811-supplementary.pdf]

Terc gene cluster variants predict liver telomere length in mice

SUPPLEMENTARY MATERIALS

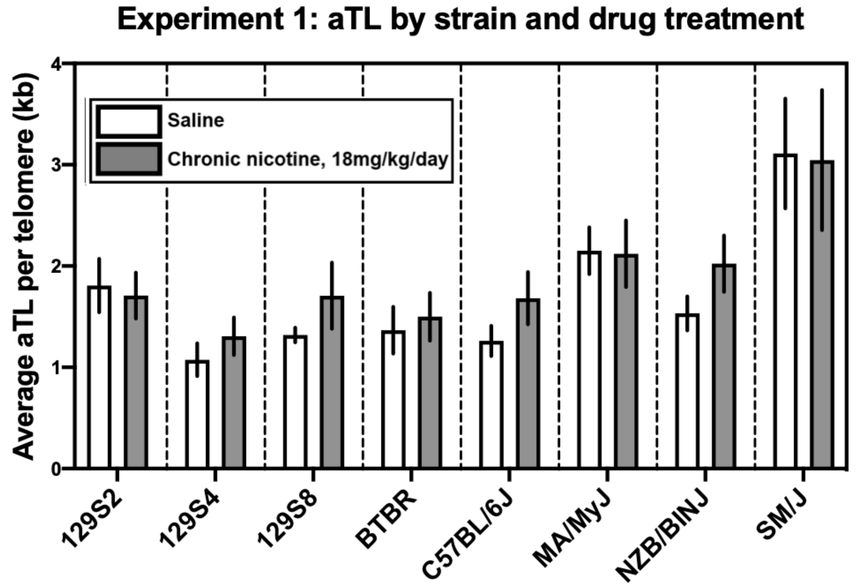

Figure S1. Average liver aTL per telomere (kb) in Experiment 1 inbred mouse strains, shown by drug treatment group. n=7-9 per tx group per strain.

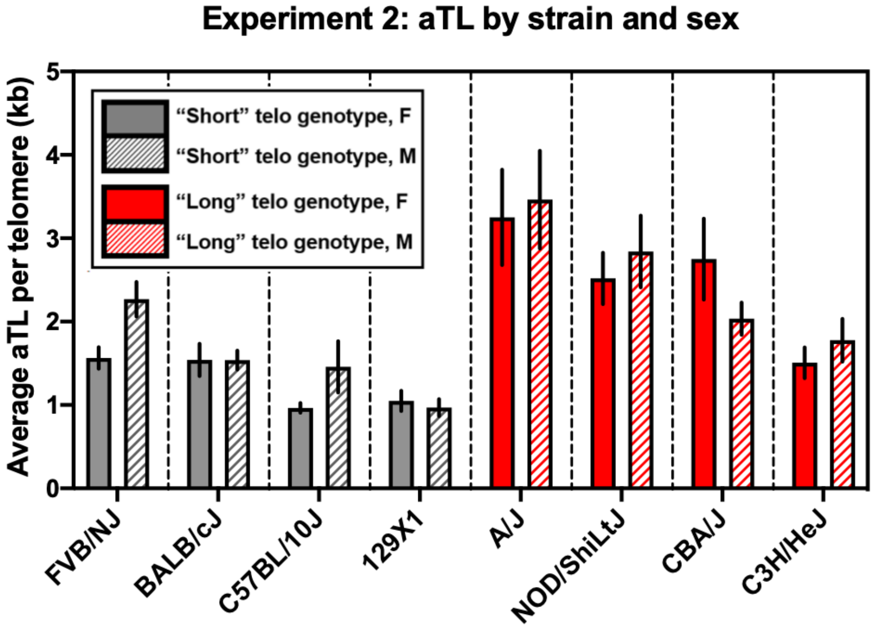

Figure S2. Average liver aTL per telomere (kb) in Experiment 2 inbred mouse strains, shown by strain and sex. n=4-7 per sex per strain.

S.1 Fear conditioning

Experiment 1 subjects were a part of a larger project testing effects of nicotine exposure and genetic background on fear conditioning. Thus, subjects underwent a two-day fear conditioning assay ending one day prior to euthanasia for liver dissections. Briefly, animals were first trained in a 5.5-minute trial consisting of two cue (30-second 85 dB white noise)/footshock (0.45mA) pairings, as previously described [41]. Twenty-four hours following training, animals were first tested for contextual fear learning in the training chamber over a 5.5-minute trial. No footshocks or auditory stimuli were administered for contextual testing. At least one hour following contextual testing, animals were placed in a distinct chamber for testing of cued fear learning over a 6-minute trial. No footshock was administered for cued testing.

S.2 Drug exposure

Experiment 1 subjects were chronically exposed to 18 mg/kg/day nicotine (freebase dose) or saline via a subcutaneously implanted osmotic minipump (model #1002, Alzet Inc.; Cupertino, CA, USA). Surgical implantation and removal of the mini pumps were performed under 3.5% isoflurane anesthesia using aseptic procedures, as described previously [41,42]. Pumps were removed 12 days following implantation and one day prior to the fear conditioning assay.

S.3 Genotyping

Because genotype information was unavailable for two of the tested strains (129S2/SvPasCrl and 129S8/SvEvNimrJ) from Experiment 1, these strains were genotyped at 6 of 7 of the candidate SNPs (rs30949246, rs31243894, rs31276550, rs30806081, rs30896355, and rs31590416, Table S1). No working primer sets could be identified for SNP rs31382064 (located within *Lrrc31*). DNA extracted from liver using the Qiagen DNeasy Blood and Tissue Kit (Hilden, Germany) was used for genotyping PCR. Primer sequences used for PCR amplification are shown in Table S1. Specifically, sequences surrounding candidate SNPs were PCR amplified and isolated on a 1% agarose gel. DNA was extracted from gel isolates using the QiaQuick Gel Extraction Kit (Qiagen, Hilden, Germany) and Sanger sequenced by the Penn State Genomics Core to identify alleles at each candidate SNP.

Primer sequences	SNP	Gene	Product size (bp)	Start alignment site (Chromosome 3, bp)
**F**: CCTGTGTGTGTGTGATTCGT<br>**R**: GTTTACAGGGTGCTTTTGTTTG	rs30806081	*Lrrc31*	244	30691351
**F**: TGCAAGTTTAATGCTGGTTATTG<br>**R**: AGGGCTCTTCAAAGACGAAA	rs31243894	*Lrrc31*	228	30679752
**F**: ACAGACCTGAAAGTTGTGGGA<br>**R**: ATCTCTCTCTAGGGCGGACT	rs31276550	*Lrrc31*	116	30682997
**F**: TGTTTCCTGAACATGCCATATC<br>**R**: TTGGATCATTACAGCATTGTACC	rs30896355	*Lrriq4*	225	30657916
**F**: TCCCTTCATGTTCCTCCCTC<br>**R**: GGACAATCACGCTCCAATCC	rs31590416	*Lrriq4*	238	30660201
**F**: TGCATCATGGTGAGGAAAAA<br>**R**: GGTGGACAAGCATCCAACTC	rs30949246	*Mynn*	240	30609060

Table S1. Genotyping primer information.
